# Sarcopenia and Sarcopenic Obesity on Body Composition Analysis is a Significant Predictor of Mortality in Severe Acute Pancreatitis: A Longitudinal Observational Study

**DOI:** 10.1007/s00268-023-07122-1

**Published:** 2023-08-04

**Authors:** Robert Farquhar, Scott Matthews, Nesta Baxter, George Rayers, Chathura B. B. Ratnayake, Francis P. Robertson, Sandip Nandhra, Wei Boon Lim, Miles Witham, Sanjay Pandanaboyana

**Affiliations:** 1 School of Medical Education, Newcastle University, Newcastle Upon Tyne, UK; 2 Department of Surgery, Royal Victoria Infirmary, Newcastle Upon Tyne, UK; 3 Department of Surgery, The University of Auckland, Auckland, New Zealand; 4 HPB and Transplant Unit, Freeman Hospital, Newcastle Upon Tyne, UK; 5 Population Health Sciences Institute, Newcastle University, Newcastle Upon Tyne, UK

## Abstract

**Background:**

The prevalence and impact of sarcopenia and sarcopenic obesity noted on body composition analysis in severe acute pancreatitis (SAP) is unknown. This study investigates the prevalence of sarcopenia at different timepoints and its effect on post-pancreatitis complications and mortality.

**Methods:**

A prospective database of SAP admissions with organ failure at a single institution from 2015 to 2019 were analysed. Sarcopenia was determined by IMAGE J software on CT. Database was further queried for post-pancreatitis complications and mortality.

**Results:**

141 patients with a median age of 59 (range 18–88) and M:F ratio 1.52:1 of were analysed. Sarcopenia was present in 111/141 (79%) patients at admission, 78/79 (99%) at 3 months and 26/36 (72%) at 12 months. 67/111 patients with sarcopenia on admission had sarcopenic obesity. The mortality at 30 days, 3 months and 12 months was 16/141 (11%), 30/141 (21%) and 42/141 (30%) respectively. Mortality was significantly higher in sarcopenic patients at admission (35.14%) compared to the non-sarcopenic group (10%), *P* = 0.008). Mortality in the sarcopenic obesity group was significantly higher (45%) compared to the sarcopenic non-obese group (20%), *P* = 0.009) at admission. Multivariate logistic regression identified sarcopenic obesity (OR: 2.880), age (OR: 1.048) and number of organ failures (OR: 3.225) as significant predictors of mortality.

**Conclusions:**

Sarcopenia and Sarcopenic obesity are highly prevalent in SAP patients on admission and during follow up. Furthermore, sarcopenic obesity was shown to be a significant predictor of mortality at admission, suggesting that body composition analysis could be a potential predictive marker of mortality in SAP patients.

**Supplementary Information:**

The online version contains supplementary material available at 10.1007/s00268-023-07122-1.

## Introduction

The incidence of acute pancreatitis (AP) in the United Kingdom has more than doubled in the past three decades, accounting for 20,000 hospital admissions a year [1]. Whilst most patients will have a mild, self-limiting episode, 20–30% of patients develop severe acute pancreatitis (SAP) with mortality of up to 50% [2]. Prediction of severity and mortality in AP is often difficult, with the current prediction scoring systems having low specificity with poor prognostic accuracy [3]. In addition, current radiological scoring systems in use for AP predominantly focus on pancreatitis related complications during the early stage of the disease process and do not predict long term outcomes.

Previous studies have shown that SAP has a significant impact on long term quality of life (QOL), affecting general health and vitality domains compared to healthy controls [4]. A significant proportion of patients have an impaired physical component of QOL at 12 months [5]. Prolonged hospital stay and reduced physical mobility likely has an effect on long term recovery of physical function [5]. Since the publication of Revised Atlanta Criteria (RAC) in 2012 [6], Computed Tomography (CT) imaging is routinely undertaken in patients with SAP throughout hospital admission to assess pancreatitis severity and screen for complications. This has allowed assessment of body composition and its impact on severity of AP and mortality. Several studies in the recent past have shown that muscle and adipose tissue measurements in AP influence prognosis, particularly sarcopenia [7–9]. Body composition parameters such psoas muscle area (PMA), total abdominal muscle area (TAMA), skeletal muscle attenuation (SMA), skeletal muscle index (SMI) and psoas muscle index (PMI) can be calculated from CT images. This allows the identification of sarcopenia and sarcopenic obesity at admission and throughout a patients stay in hospital and recovery, a method validated by the European Working Group on Sarcopenia in Older People (EWGSOP) [10]. Although several studies [8, 11] and a recent systematic review confirmed the utility of body composition analysis on short term outcomes in AP, there is no published data on temporal trends on body composition in patients with SAP, particularly in those admitted to Intensive Treatment Units (ITU) with organ failure. The present study aims to investigate the prevalence of sarcopenia in SAP patients based on body composition analysis at admission, 6 weeks, 3 months, 6 months, and 12 months post-admission to determine the effect of sarcopenia on post-pancreatitis complications and mortality. In addition, the effect of sarcopenic obesity on post-pancreatitis outcomes was further evaluated.

## Methods

### Subjects and study design

Patients were identified from a prospectively maintained database of SAP patients at the Freeman Hospital, Newcastle upon Tyne, in the Northeast of England. The Freeman Hospital is a regional specialist centre for SAP patients and with an established hub and spoke pathway [12].

The management of pancreatic necrosis in our institution is based on a step-up approach strategy with laparotomy very rarely undertaken [13]. Organ failure was defined based on the assessment of the 3 organ systems i.e. respiratory, cardiovascular, and renal. Organ failure is defined as score of 2 or more for one of these three organ systems using the modified Marshall scoring system [14]. Patients with organ failure were managed in the ITU with respiratory (artificial ventilation), renal (dialysis) and cardiovascular support when needed. Complications of SAP, for example walled of necrosis and pseudoaneurysms, were treated as they arise with endoscopic or interventional radiology support. As the inflammation from the pancreatitis settles and the patient no longer requires organ support, they were discharged to a specialist ward to further rehabilitation.

The patient’s nutritional needs were managed by dieticians with regular dietetic follow up in the ITU and on the wards. Patients were encouraged oral nutrition on admission. If oral nutrition could not be tolerated, nasogastric or nasojejunal feeding was the preferred feeding route with total parenteral nutrition (TPN) considered for patients who could not tolerate enteric feeding. Pancreatic enzyme replacement was routinely prescribed. After discharge, patients had ongoing surgical clinical follow up for 12 months in outpatient surgical clinics. Dietetic assessment was only provided where it was needed i.e., poor oral intake warranting enteric feeding at home and patients were readmitted to the hospital if there were concerns for weight loss at routine outpatient appointments. Formal follow up physiotherapy appointments were not offered.

The prospectively collected data included baseline characteristics such as age, sex, body mass index (BMI) on admission, smoking status, and the aetiology of AP. ITU stay, the total duration of ITU stay(s) and whether the patient had renal and/or respiratory failure was recorded.

Other complications were identified, including the presence of walled off necrosis or pseudocyst, pseudoaneurysms, pancreatic duct disruption, pancreatic ascites, pancreatic-pleural fistula, enteric fistula, and portal vein thrombus. Mortality, including the underlying causes was also recorded, determined from hospital records, autopsy reports or death certificates.

Data regarding the method of feeding and pancreatic enzyme replacement usage was recorded. The use of endoscopic, percutaneous, or open necrosectomy were also recorded, as were interventional radiology treatments if warranted, primarily used to manage bleeding pseudoaneurysms.

The development of diabetes mellitus (DM) secondary to SAP, readmission with AP, cholecystectomy post-discharge and the number of follow-up appointments the patient attended were all recorded.

### Definitions

Diagnosis of SAP and terminology of complications was based on RAC definition [6].

Obesity was defined in line with the WHO definition [15] of a BMI >30 kg/m^2^. Where height and/or weight data was unavailable (10% of patients), waist circumference was used as a surrogate measure of obesity. Waist circumference was measured at the third lumbar vertebrae (L3) level of a CT scan, with obesity then defined in line with the literature, as a waist circumference measurement of >102 cm in men and >88 cm in women [16].

Sarcopenia was defined based on previous validated CT derived sarcopenia thresholds for SMI, PMI and SMA. For SMI, this threshold is <43 cm^2^/m^2^ for men with a BMI <25, and a SMI of <53 cm^2^/m^2^ in men with BMI >25. In women, an SMI of <41 cm^2^/m^2^ was used as per the definition of Martin et al. [17]. A PMI of <5.9 cm^2^/m^2^ was used to define sarcopenia in men and <4.1 cm^2^/m^2^ in women as per the definition of Okumura et al. [18]. A SMA measurement of <33.9 HU in men and <30.9 HU in women would be used as a threshold for sarcopenia, as per the definition of Dijk et al. [19].

Sarcopenic obesity was defined as a person meeting one of the 3 sarcopenia thresholds and in addition being classed as obese, either via a BMI >30 kg/m^2^ or waist circumference >102 cm in men and >88 cm in women [20, 21].

### CT scan analysis of sarcopenia and sarcopenic obesity

All patients with SAP had a CT scan within 7 days of admission to assess the severity of pancreatitis.

Axial images from the superior endplate of the L3 vertebrae were identified [22]. Assessment was carried out using NIH ImageJ v1.53e software (Laboratory for Optical and Computational Instrumentation, University of Wisconsin, Madison, WI, USA) [23].

PMA was calculated by tracing the psoas muscles, then applying a validated Hounsfield unit (HU) threshold for values typical of skeletal muscle (−29 to +150 HU). TAMA was calculated by excluding all structures both external and internal to the abdominal musculature and applying the same HU threshold. The mean HU of the abdominal musculature gave the measurement for SMA.

To contextualise these measurements for each patient, the TAMA and PMA measurements were then divided by the squared height of each individual patient. This enabled the calculation of the SMI and PMI respectively. CT scans were collected from 5 discrete time points during the patient’s hospital stay and recovery, where available. These included at admission, 6 weeks, 3- 6- and 12-months post admission.

### Statistical analysis

All data was analysed using IBM SPSS statistics software (Version 27.0. Armonk, NY: IBM Corp) [24]. Continuous data was presented as mean ± SD or median (interquartile range). Nominal data was analysed using Chi-squared tests, or Fisher’s exact tests if an expected value was <5. Data is displayed as number (percentage). Quantitative data was analysed using Mann–Whitley U-tests. Data is expressed as median (range). A result was statistically significant if a *P*-value of <0.05 was achieved.

Potential predictors of mortality in SAP patients were first identified by univariate analysis, with four factors of interest with a *P*-value < 0.05 chosen to be included in a multivariate logistic regression model.

All scans were initially analysed by RF and then independently re-analysed by either SM or NB, both of whom were blinded to the patient’s information. Pearson’s correlation coefficient was then calculated to ensure accuracy been the two analyses. A value of 0.73 was calculated, suggesting a high level of correlation between the two analyses.

## Results

### Baseline characteristics and organ failure

A total of 141 patients were diagnosed with SAP and admitted to ITU during the study period (02/01/2015–29/12/2019). The median age was 59 years (IQR 48–70), and a M:F ratio of 1.52:1. The median BMI was 29.68 (IQR 24.80–32.76), with 78/141 (55%) of patients classified as obese (BMI > 30). 139/141 (99%) of patients had respiratory failure and 78/141 (55%) of patients had renal failure. 78/141 (58%) of patients had >1 organ failure.

### Temporal trends in sarcopenia and sarcopenic obesity based on body composition parameters

**Fig. 1:**
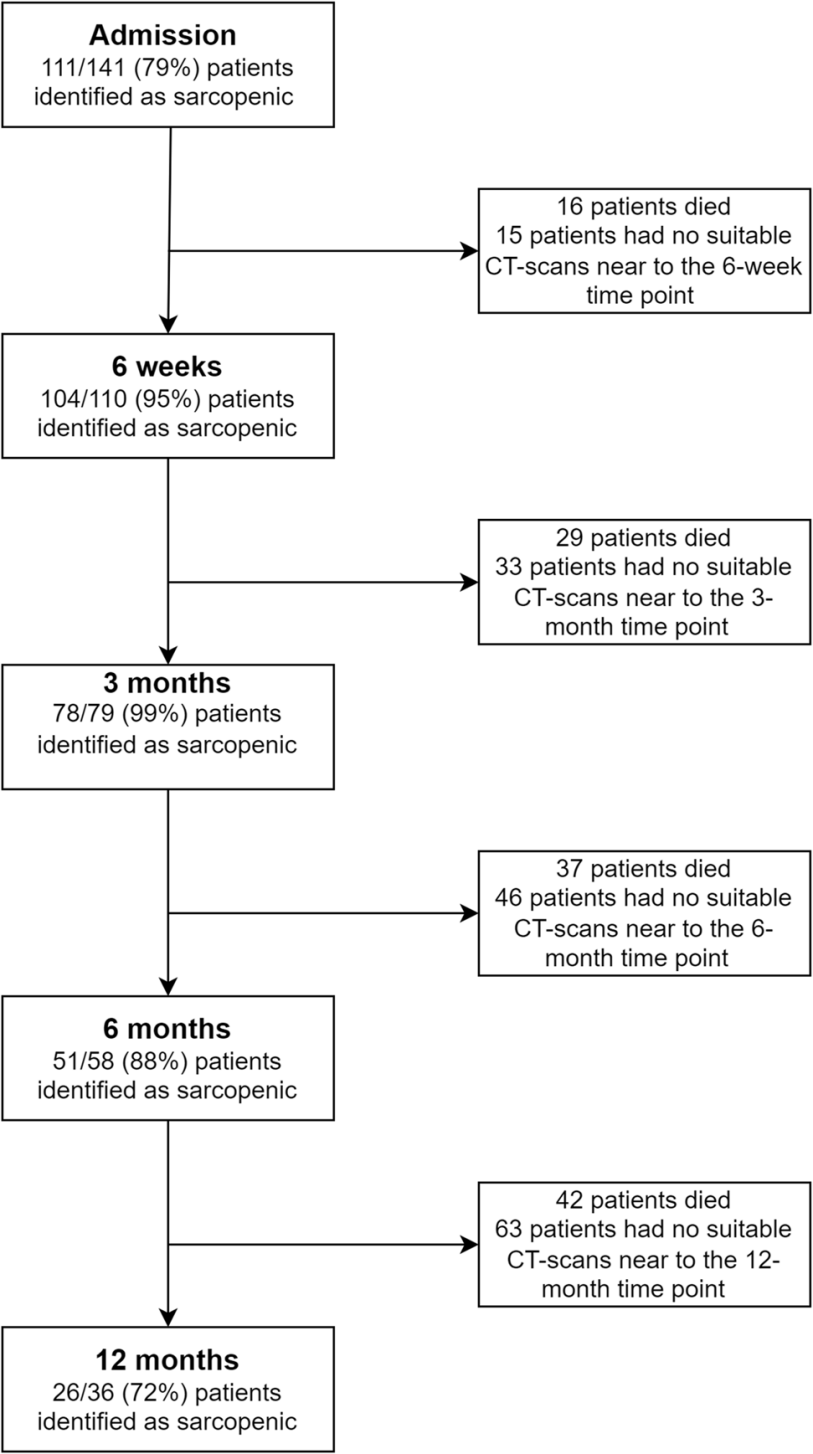
Flow chart summarising mortality and proportion of patients with Sarcopenia at admission, 6 weeks, 3 months, 6 months, and 12 months post admission

On admission, 111/141 patients (79%) were sarcopenic based on the CT body composition parameters. At 6 weeks, the proportion of sarcopenic patients increased to 104/110 (95%), increasing further at 3 months to 78/79 patients (99%). At 6 months, 51/58 patients were sarcopenic (88%) and at 1 year post admission 26/36 patients (72%) were sarcopenic. Of the 111 patients presenting with sarcopenia on admission, 67 (60%) were classified as having sarcopenic obesity. 5/26 (19%) sarcopenic patients were sarcopenic obese at 12-month post-discharge. There has been attrition of patients during follow up from mortality and lack of follow up CT scans, summarised by Fig. 1.

### Comparison of sarcopenia, non-sarcopenia and sarcopenic obesity groups

When comparing the sarcopenia versus non-sarcopenia groups, patients in the sarcopenia group were significantly older [62 (50–72), vs. 49 (32.25–62) *P* < 0.001] and more obese (60.3% vs. 36.6% *P* = 0.021). Hypertension and CKD were more common in the sarcopenic group. When comparing the sarcopenia versus sarcopenic obese groups, the sarcopenic obese group were significantly older (64 years vs. 55 years, *P* = 0.009). The sarcopenic obese group were found to have significantly higher rates of hypertension [30/67 (45%) vs. 10/44 (22.73%), *P* = 0.018] and diabetes mellitus [18/67 (27%) vs. 5/44 (11%), *P* = 0.049] versus the sarcopenia group. Gallstone disease was a more common aetiology in the sarcopenic obese group [33/67 (49%)] compared to the sarcopenia group [8/44 (18%), *P* = 0.001; Table 1].

**Table 1:** Baseline characteristics, comorbidities, and body composition parameters between the SARC and NSARC groups

Variable	Sarcopenic group (n = 111)	Non-sarcopenic group (n = 30)	*P* value
Age (years), median (IQR)	62 (50–72)	49 (32.25–62)	**<0.001**
Male sex, n (%)	65 (59%)	20 (67%)	0.421
Body mass index (kg/m^2^), median (IQR)	30.3 (24.7–33.6)	27.9 (26.0–31.5)	0.285
Obesity, n (%)	67 (60%)	11 (37%)	**0.021**
Previous cholecystectomy, n (%)	16 (14%)	5 (17%)	0.775
Previous acute pancreatitis, n (%)	18 (16%)	3 (10%)	0.566
Chronic pancreatitis, n (%)	11 (10%)	2 (7%)	0.735
COPD^a^, n (%)	8 (7%)	2 (7%)	1.000
Asthma, n (%)	7 (6%)	4 (13%)	0.247
Ischaemic heart disease, n (%)	14 (13%)	3 (10%)	1.000
Hypertension, n (%)	40 (36%)	5 (17%)	**0.043**
Chronic kidney disease, n (%)	14 (12%)	0	**0.041**
Cerebral vascular disease, n (%)	6 (5%)	0	0.342
Diabetes mellitus, n (%)	23 (21%)	4 (13%)	0.362
PMI^b^ on admission scan (cm^2^/m^2^)	4.30 (3.51–5.42)	6.45 (5.89–7.19)	**<0.001**
SMI^c^ on admission scan (cm^2^/m^2^)	46.83 (38.80–54.35)	57.06 (50.43–66.04)	**<0.001**
SMA^d^ on admission scan (HU)	22.67 (18.26–28.08)	37.40 (34.33–40.55)	**<0.001**
Gallstone aetiology	41 (37%)	14 (47%)	0.332
Alcohol aetiology	31 (28%)	9 (30%)	0.823

Statistically significant with a *P* value of <0.05 are given in bold

^a^Chronic obstructive pulmonary disease

^b^Psoas muscle index

^c^Skeletal muscle index

^d^Skeletal muscle attenuation

### Post-pancreatitis complications and length of stay between sarcopenia, non-sarcopenia and sarcopenic obesity groups

Acute pancreatic fluid collections, pseudoaneurysms warranting radiological embolisation, need for endoscopic necrosectomy and laparotomy for complications were more common on the non-sarcopenia group.

Overall mortality on the other hand was higher in the sarcopenic obesity group. The length of ITU stay and overall length of hospital stay was similar between all groups (Table 2).

**Table 2:** Post-pancreatitis complications, length of stay and readmission between NSARC, SARC and SARC obese groups

Variable	Non-sarcopenic group (n = 30)	Sarcopenic group (n = 44)	Sarcopenic obese group (n = 67)	*P* value
Length of hospital stay (days), median (range)	103 (251–20)	74 (229–23)	91 (270–14)	0.520
Length of ITU stay (days), median (range)	31 (109–5)	16 (84–1)	20 (117–1)	0.087
Drainage of pancreatic collection, n (%)	25 (83%)	33 (75%)	42 (63%)	0.091
Necrosectomy, n (%)	22 (73%)	28 (64%)	29 (43%)	**0.011**
Treatment with Interventional radiology, n (%)	9 (30%)	5 (11%)	7 (10%)	**0.032**
ERCP^a^, n (%)	9 (30%)	12 (27%)	13 (19%)	0.445
Laparotomy, n (%)	5 (17%)	6 (14%)	2 (3%)	**0.047**
New onset of diabetes mellitus, n (%)	9/27 (33%)	9/35 (26%)	11/37 (30%)	0.805
Readmission with acute pancreatitis, n (%)	6/27 (22%)	9/35 (26%)	7/37 (19%)	0.786
Readmission other, n (%)	13/27 (48%)	14/35 (40%)	12/37 (32%)	0.444
Cholecystectomy post-discharge, n (%)	10/27 (37%)	8/35 (23%)	11/37 (30%)	0.476
Acute Pancreatic Fluid Collections, n (%)	30 (100%)	44 (100%)	59 (88%)	**0.009**
Walled off necrosis, n (%)	27 (90%)	32 (75%)	42 (63%)	**0.022**
Pseudoaneurysm, n (%)	10 (33%)	5 (11%)	5 (7%)	**0.003**
Duct disruption, n (%)	1 (3%)	7 (16%)	2 (3%)	**0.023**
Pleural fistula, n (%)	1 (3%)	1 (2%)	0	0.372
Enteric fistula, n (%)	7 (23%)	5 (11%)	8 (12%)	0.269
Portal vein thrombus, n (%)	10 (33%)	10 (23%)	13 (19%)	0.323
Positive blood cultures, n (%)	8 (27%)	14 (32%)	27 (40%)	0.379
Mortality, n (%)	3 (10%)	9 (20%)	30 (45%)	**0.001**

Statistically significant with a *P* value of <0.05 are given in bold

^a^Endoscopic retrograde cholangiopancreatography

The indications for early (<4 weeks) surgical intervention were splenic rupture (n = 1) and ascending colon perforation (n = 1).

The indications for late surgical intervention (4 weeks to 6 months) were: Ischaemic bowel (n = 1); treatment of bleeding from splenic artery aneurysm (n = 1); removal of gastric necrosis (n = 1); small bowel perforation (n = 1); surgical necrosectomy to drain necrotic tissue not accessible percutaneously (n = 2); bypassing a duodenal fistula (n = 1); washout to treat peritonitis secondary to perforation of the stomach following endoscopic stent placement (n = 1); duodenal exclusion to treat the source of a fistula (n = 1); treatment of abdominal wall bleeds (n = 2); bile leak (n = 1) and peritonitis (n = 1).

### Sarcopenia, sarcopenic obesity and mortality

Mortality at 30 days, 3 months and 12 months post-admission was 16/141 (11%), 30/141 (21%) and 42/141 (30%) respectively. The most common cause of mortality was multi-organ failure secondary to acute pancreatitis, causing 83% (35/42) of the deaths. Type 2 respiratory failure was the second most common (3/42 7%), with biliary sepsis, perforation of the small bowel and gastrointestinal haemorrhage all causing one death each (1/42, 2%).

**Fig. 2:**
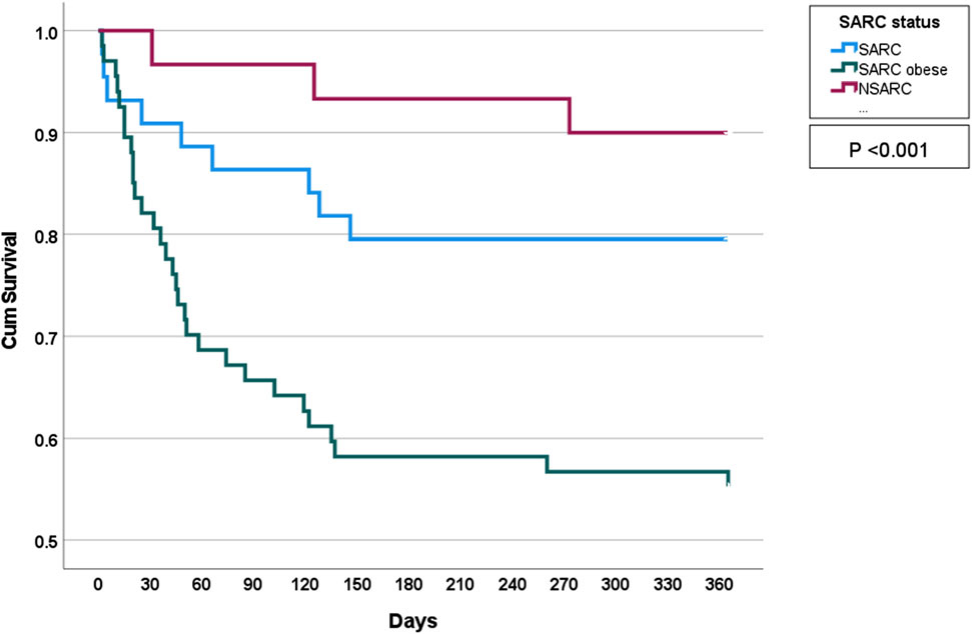
KM Curve of survival analysis of Non-sarcopenic (NSARC), sarcopenic (SARC) and sarcopenic obese patients

As seen in Supplementary File 1, mortality was significantly higher in the sarcopenia group [39/111 (35.14%)], compared to the non-sarcopenia group [3/30 (10.00%), *P* = 0.008]. Mortality was significantly higher in the sarcopenic obese group [30/67 (45%)] compared to the sarcopenia group (9/44 (20%) *P* = 0.009). This difference in mortality is visualised in the Kaplan-Meier survival graph below (Fig. 2).

On univariate analysis age, obesity, a past medical history of hypertension and chronic kidney disease, the number of organ failures, presence of renal failure, sarcopenia on admission and sarcopenic obesity on admission were identified as potential predictors of mortality. Multivariate logistic regression identified age, the number of organ failures and sarcopenic obesity on admission as significant predictors of overall mortality, as seen in Supplementary Table 1.

### Follow-up

A higher proportion of the non-sarcopenic patients were readmitted to hospital for a reason other than AP [non-sarcopenia—13/27 (48%) vs. sarcopenia—26/72 (36%), *P* = 0.275] and had a cholecystectomy in the follow up period [Non-sarcopenia—10/27 (37%) vs. sarcopenia—19/72 (26%), *P* = 0.300] compared to the sarcopenia group; however, these differences were not statistically significant.

## Discussion

The results of this study have shown that sarcopenia (79%) and sarcopenic obesity (48%) are highly prevalent in patients admitted to ITU with severe acute pancreatitis. The prevalence of sarcopenia peaked 3 months into admission (99%) and remained high at 12 months (72%). On multivariate analysis, the presence of sarcopenic obesity, advanced age and multi-organ failure were independent predictors of overall mortality in SAP.

Sarcopenia, radiologically defined using CT parameters, was highly prevalent in patients admitted to our unit with SAP (79%). Although all these patients were started on supplementary enteral or parenteral feed, the prevalence of sarcopenia increased throughout the stay and was evident in 99% of patients at 3 months highlighting the impact that SAP has on the muscle mass of patients. At 1 year post admission, 72% of surviving patients who had an available CT scan to analyse remained sarcopenic; highlighting the long-term impact of the disease.

To the best of our knowledge, we are the first group to perform such a longitudinal observation study identifying the temporal changes seen in body composition in patients admitted to ITU with SAP. We found a related study by Levy et al. investigating the incidence of sarcopenia at 3 months and 6 months in COVID-19 patients admitted to the ITU [25]. They found that 16% of patients were sarcopenic at 3 months, dropping to 4% at 6 months. This is encouraging in that it shows that the reversal of sarcopenia is possible. The incidence of sarcopenia was likely higher in our study because patients with SAP typically spend longer periods of time in the ITU, and the prolonged inflammation in SAP causes significant muscle wastage.

Sarcopenic obesity was found to be highly prevalent on admission (48%) and the incidence of sarcopenia and sarcopenic obesity were significantly higher in the mortality group compared to the surviving group on univariate analysis. On multi-variate analysis only sarcopenic obesity was an independent predictor of 12-month mortality [2.88 (1.180–7.033). *P* = 0.020]. Conversely, the post-pancreatitis complications such as acute pancreatic fluid collections and pseudoaneurysms were higher in the non-sarcopenic group. We believe this is because these patients had lower early mortality and therefore longer survival with a higher risk of developing post-pancreatitis complications.

Research investigating the impact and incidence of sarcopenia in various diseases is ever expanding. Sarcopenia is commonly seen in chronic disease, for example in chronic renal disease [26, 27], type two diabetes mellitus [28] and chronic liver diseases [29]. Sarcopenic obesity has recently been shown to be a significant risk factor for postoperative morbidity after pancreatic surgery [30]. The impact of sarcopenia and sarcopenic obesity in SAP remains unclear. In a study from China, patients with AP had significantly higher visceral and subcutaneous adipose tissue and significantly lower skeletal muscle attenuation than control patients [8]. Patients with SAP were seen to have significantly more visceral adipose tissue and significantly lower skeletal muscle attenuation, which was associated with increased 3-month mortality [HR: 10.500 (1.344–82.025), *P* = 0.025]. These results, however, must be interpretated with caution as patients with severe disease had low skeletal muscle attenuation and this increased mortality rate may simply reflect the severity of disease rather than any causation. Similar results were seen in an American study of 507 patients with necrotising pancreatitis, where lower skeletal muscle density was associated with increased disease severity [31]. A further study from South Korea has similarly shown the development of moderately severe pancreatitis/SAP was associated with lower skeletal muscle volume and higher visceral fat area [9].

Whilst the results of our study are broadly in keeping with previous studies, our study only includes patients with SAP. This allows us to draw a strong conclusion from a homogeneous population that sarcopenia and sarcopenic obesity are associated with increased mortality. There are several reasons for this association. Previous studies have shown that visceral adiposity results in a chronic inflammatory state [32, 33]. Intra-pancreatic fat and visceral adipose tissue are enriched with pro-inflammatory cytokines (IL-6, TNF-a), and reduce the number of anti-inflammatory markers such as adiponectin. Adiponectin plays a crucial role in reducing the inflammatory response by inhibiting macrophage production [34–36]. Recent data into cytokine signatures in the early phase of AP are shown to be driven predominantly by IL-6, which is abundant in pancreatic and visceral fat [37]. This pro-inflammatory response in patients with excess pancreatic and peripancreatic fat may result in a severe cytokine storm, thereby aggravating the inflammatory response and worsening the severity of the AP. The current series had a high prevalence of sarcopenia at admission however, most of these patients had sarcopenic obesity (60%). Patients with sarcopenic obesity are more likely to have increased pancreatic and peripancreatic fat which may increase the risk of developing SAP from the aforementioned cytokine storm.

Our study suggests that sarcopenia increases the risk of mortality for patients admitted to the ITU with SAP. This correlates with other work exploring this relationship, which has investigated the impact of sarcopenia in ITU patients more generally. For example, a study by Weijs et al. showed that low skeletal muscle area was a risk factor for mortality in mechanically ventilated critically ill patients, regardless of the condition that had caused them to be admitted to the ITU [38]. Admission to an ITU causes muscle catabolism due to the long sedentary nature of the stay. Kortebein et al. showed that just 10 days of bed rest was sufficient time for a patient to lose a significant amount of skeletal muscles mass [39]. If a patient has a low muscle mass to begin with, the pathophysiological processes and the physiological stress induced by being in the ITU paired with a catabolic disease such as SAP depletes this further, reducing the physiological reserve and contributing to increased mortality.

Our study design has several strengths and some limitations. We assessed a small but specific group of critically ill patients using validated assessment tools and an investigator blinded to the patient’s previous history. We undertook multiple assessments, at pre-defined time intervals, in over 80% of eligible patients. The sarcopenia assessments were undertaken by 3 different investigators with excellent concordance.

The Limitations of our study included the retrospective nature of the sarcopenia assessment. This meant that we could define sarcopenia only using body composition analysis—a quantitative method, and not a qualitative method of measuring the functional strength of the muscle, where using both would have been ideal. Also, not all patients underwent CT scans at the predefined time points for sarcopenia assessment, resulting in attrition of patient numbers during follow-up.

Future studies should evaluate sarcopenia based on the ESGSOP [10] definition which not only incorporates muscle quantity based on body composition analysis but also muscle strength based on hand grip strength measurement to confirm clinical sarcopenia. In addition, Quality of life and PROMS, particularly sarcopenia specific such as SarQol [40], should be gathered to investigate how sarcopenia impacts patients QOL after SAP.

Post discharge, robust nutritional programme is warranted during follow up with expert Dietetic input. This should include clinical assessment for adequate use of PERT and appropriateness of timing of PERT and dosage in addition to assessment of malnutrition with malnutrition universal assessment tool (MUST). A full blood work monitoring for micronutrient deficiencies including regular HBA1c check and blood glucose monitoring. Finally, anthropometric assessments to check weight, BMI and Hand grip strength longitudinally will identify patients at risk of malnutrition during follow up who could benefit from hospital admission. Nutritional rehabilitation should be undertaken in conjunction with physical rehabilitation ensuring ongoing physiotherapy appointment post discharge until sarcopenia is resolved.

### Supplementary Information

Below is the link to the electronic supplementary material.

Supplementary file1 (DOCX 24 kb)
