# Supplementary material for: Sarcopenia and Sarcopenic Obesity on Body Composition Analysis is a Significant Predictor of Mortality in Severe Acute Pancreatitis: A Longitudinal Observational Study
Source: World J Surg. 2023 Aug 4;47(11):2825–33. doi: 10.1007/s00268-023-07122-1 (PMC10545625; doi:10.1007/s00268-023-07122-1)
Supplement: Supplementary file 1 — Supplementary file1 (DOCX 24 kb) [file 268_2023_7122_MOESM1_ESM.docx]

| **Potential predictor of mortality** | **Mortality group (n=42)** | **Surviving group (n=99)** | **Univariate P-value** |
| --- | --- | --- | --- |
| Age (years), median (IQR) | 69 (59.25 - 74.75) | 54 (43 - 65.6) | **<0.001** |
| Male sex, n (%) | 23 (55%) | 62 (63%) | 0.383 |
| Obesity, n (%) | 30 (71%) | 46 (51%) | **0.007** |
| Body mass index MI (kg/m²), median (IQR) | 30.6 (27.2 - 34.5) | 28.82 (24.5 - 32.5) | 0.054 |
| Smoking status, n (%) | 16 (76%) | 52 (53%) | 0.162 |
| Previous laparoscopic cholecystectomy, n (%) | 4 (10%) | 17 (17%) | 0.243 |
| Previous acute pancreatitis, n (%) | 6 (14%) | 15 (15%) | 0.895 |
| Chronic pancreatitis, n (%) | 3 (7%) | 10 (10%) | 0.755 |
| Chronic obstructive pulmonary disease, n (%) | 3 (7%) | 7 (7%) | 1.000 |
| Asthma, n (%) | 0 | 11 (11%) | **0.034** |
| Ischaemic heart disease, n (%) | 8 (19%) | 9 (9%) | 0.097 |
| Hypertension, n (%) | 21 (50%) | 24 (24%) | **0.003** |
| Chronic kidney disease, n (%) | 9 (21%) | 4 (4%) | **0.002** |
| Cerebral vascular disease, n (%) | 4 (10%) | 2 (2%) | 0.065 |
| Diabetes mellitus, n (%) | 10 (23%) | 17 (17%) | 0.360 |
| Amylase on admission, n (%) | 1470 (726 - 2245) | 1004 (300 - 1886) | 0.129 |
| Bilirubin on admission, n (%) | 14 (9.8 - 36.3) | 20 (10.5 - 50) | 0.279 |
| Gallstone aetiology, n (%) | 16 (38%) | 39 (39%) | 0.125 |
| Alcohol aetiology, n (%) | 8 (19%) | 32 (32%) | 0.501 |
| Post-ERCP aetiology, n (%) | 3 (7%) | 12 (12%) | 0.425 |
| Transferred from district general hospital, n (%) | 26 (62%) | 59 (59%) | 0.798 |
| Number of organ failures, n (%) | 1.83 (SD - 0.37) | 1.41 (SD - 0.53) | **<0.001** |
| Respiratory failure, n (%) | 42 (100%) | 97 (97%) | 1.000 |
| Renal failure, n (%) | 35 (83%) | 43 (43%) | **<0.001** |
| Collection, n (%) | 39 (93%) | 94 (96%) | 0.695 |
| Pseudoaneurysm, n (%) | 5 (12%) | 15 (15%) | 0.613 |
| Duct disruption/pancreatic ascites, n (%) | 4 (10%) | 6 (6%) | 0.484 |
| Pancreatic-pleural fistula, n (%) | 0 | 2 (2%) | 1.000 |
| Enteric fistula, n (%) | 8 (19%) | 12 (12%) | 0.281 |
| Portal vein thrombus, n (%) | 8 (19%) | 25 (25%) | 0.426 |
| Positive blood cultures, n (%) | 18 (43%) | 31 (31%) | 0.188 |
| Sarcopenia on admission, n (%) | 39 (93%) | 72 (74%) | **0.008** |
| Sarcopenic obesity on admission, n (%) | 30 (71%) | 37 (37%) | **<0.001** |
| PMI* on admission scan (cm²/m²) | 24.5 (16.0 - 29.0) | 26.99 (19.9 - 34.1) | 0.050 |
| SMI** on admission scan (cm²/m²) | 4.87 (3.52 - 6.18) | 4.95 (4.03 - 6.12) | 0.723 |
| SMA^??^ on admission scan (HU) | 47.30 (40.1 - 54.4) | 50.43 (41.0 - 57.3) | 0.374 |

|  | Multivariate analysis | | | |
| --- | --- | --- | --- | --- |
| Potential predictors of mortality | Odds ratio (Exp B) | Standard error | 95% CI for Exp (B) | P-value |
| Age | 1.048 | 0.017 | 1.021 - 1.095 | **0.005** |
| Number of organ failures | 3.225 | 0.38 | 1.545 - 6.857 | **0.002** |
| SARC obesity | 2.88 | 0.455 | 1.180 - 7.033 | **0.020** |
| Chronic kidney disease | 2.71 | 0.669 | 0.730 - 10.055 | 0.136 |

**Supplementary file 1: Predictors of overall mortality on univariate analysis**

*** Psoas muscle index**

****Skeletal muscle index**

**?? Skeletal muscle attenuation**

**Supplementary table 1: Predictors of overall mortality on logistic regression**
